# Identification and characterization of *Dof* genes in *Cerasus humilis*

**DOI:** 10.3389/fpls.2023.1152685

**Published:** 2023-04-03

**Authors:** Weili Liu, Weichao Ren, Xiubo Liu, Lianqing He, Chen Qin, Panpan Wang, Lingyang Kong, Yang Li, Yunwei Liu, Wei Ma

**Affiliations:** ^1^ School of Pharmacy, Heilongjiang University of Chinese Medicine, Harbin, China; ^2^ Experimental Teaching and Training Center, Heilongjiang University of Chinese Medicine, Harbin, China; ^3^ School of Jiamusi, Heilongjiang University of Chinese Medicine, Jiamusi, China; ^4^ Berry Resource Research Center, Yichun Branch of Heilongjiang Academy of Forestry, Yichun, China

**Keywords:** *Cerasus humilis*, DOF, transcription factor, fruit storage, gene expression analysis

## Abstract

**Introduction:**

*Dof* genes encode plant-specific transcription factors, which regulate various biological processes such as growth, development, and secondary metabolite accumulation.

**Methods:**

We conducted whole-genome analysis of Chinese dwarf cherry (*Cerasus humilis*) to identify *ChDof* genes and characterize the structure, motif composition, cis-acting elements, chromosomal distribution, and collinearity of these genes as well as the physical and chemical properties, amino acid sequences, and phylogenetic evolution of the encoded proteins.

**Results:**

The results revealed the presence of 25 *ChDof* genes in *C. humilis* genome. All 25 *ChDof* genes could be divided into eight groups, and the members of the same group had similar motif arrangement and intron-exon structure. Promoter analysis showed that cis-acting elements responsive to abscisic acid, low temperature stress, and light were dominant. Transcriptome data revealed that most *ChDof* genes exhibited tissue-specific expression. Then, we performed by qRT-PCR to analyze the expression patterns of all 25 *ChDof* genes in fruit during storage. The results indicated that these genes exhibited different expression patterns, suggesting that they played an important role in fruit storage.

**Discussion:**

The results of this study provide a basis for further investigation of the biological function of *Dof* genes in *C. humilis* fruit.

## 1 Introduction

*Dof* (DNA binding with one finger) genes encode plant-specific zinc finger proteins with 200-400 amino acid residues, a conserved domain consisting of 52 aa residues at the N-terminal end, and a variable domain for transcriptional regulation at the C-terminal end. Most importantly, Dof proteins contain C2-C2 type zinc finger motifs that recognize specific regulatory elements (AAAG or CTTT) in the promoters of target genes (Plesch et al., 2001; Yanagisawa, 2004; Gupta et al., 2015). The main domains are the highly conserved N-terminal DNA-binding domain and the C-terminal transcriptional regulatory domain. The amino-terminal sequence of the C-terminal transcriptional regulatory domain is variable and less conserved than its carboxy-terminal sequence, and is likely responsible for the diverse regulatory roles of Dof proteins in plant growth and development. The N- and C-terminal ends of Dof proteins interact with various regulatory proteins or intercept signals to mediate the activation or inhibition of target genes(Noguero et al., 2013). The *Dof* gene family is found in a wide variety of plant species.

Members of the *Dof* gene family are involved in plant growth and development (Fornara et al., 2009), abiotic stress (Cai et al., 2016; Yang et al., 2017), plant hormone (Kang and Singh Karam, 2000, Jin et al., 2014; Qin et al., 2019), and light response, and light signal transduction (Park et al., 2003, Kondhare et al., 2019). Some functions of Dof proteins have been confirmed to date. For example, in *Arabidopsis thaliana*, Dof transcription factors promote healing post grafting, hypocotyl extrusion, and callus formation and facilitate wound recovery after mechanical injury (Zhang et al., 2022). In tomato (*Solanum lycopersicum*), RNA interference experiment showed the expression of *SIDof1* is inhibited during fruit ripening, thus delaying the synthesis of lycopene and the process of fruit ripening (Wang et al., 2021). In cherries, in the presence abscisic acid (ABA), PavDof6 directly binds to the promoter encoding the cell wall modification enzyme gene, while PavDof2/15 plays the opposite role, confirming that some members of the *PavDof* gene family are involved in early maturation and delayed softening of the fruit (Zhai et al., 2022). Transcriptome analysis of kiwifruit showed that *Dof* genes play a key role in flower and fruit development (Brian et al., 2021). *AdDof3*, *AdDof4*, and *AdNAC5* are involved in the ripening and softening of kiwi fruit; *AdDof3* promotes kiwifruit maturation by binding to and activating the promoter of *AdBAM3L*, a key gene required for starch degradation (Zhang et al., 2018). While exploring the response of ethylene to volatile compounds in kiwifruit, we previously demonstrated that *AdDof4* trans-inhibits the promoter of a fatty acid desaturase gene, AdFAD1 (Zhang et al., 2020).

Chinese dwarf cherry (*Cerasus humilis*), which is endemic to China, is a perennial shrub of the *Cerasus* genus with high ecological and economic value. *C. humilis* shows strong resistance to abiotic stresses, especially drought and low temperature, and is therefore used for windproof sand fixation. Additionally, *C. humilis* has long been used as a traditional Chinese medicine, specifically Yu Li Ren, which is prepared from its dried seed (Wu et al., 2019). Rosaceae species such as apple and pear, which contain high amounts of calcium in their fruit, and *C. humilis* is no exception. The fruit of *C. humilis* contains the highest amount of calcium among all Rosaceae species, and is therefore known as “calcium fruit”, which is very popular among people. Given its high economic value, *C. humilis* has been widely cultivated in northern China in recent years (Wang et al., 2017; Wang et al., 2020).

Lignin, a polymer of phenylpropanol derivatives, is deposited in secondary cell walls to increase the mechanical strength of xylem in vascular plants and to defend against invading pathogens (Zhao and Dixon, 2011). Generally, the hardness of fruit is related to the lignin and cellulose content of plant cells. In sugarcane, Dof transcription factors containing C2C2 zinc finger domains regulate cellulose and lignin metabolism and act as major players in carbon metabolism (Kasirajan et al., 2018). In *Arabidopsis*, VDof1 and VDof2 regulate vascular cell differentiation throughout the life cycle of a plant by targeting different genes at different developmental stages; VDof proteins negatively regulate vein formation at the seedling stage and target lignin biosynthesis at the reproductive stage (Ramachandran et al., 2020). Previous studies have shown that members of the *Dof* gene family play a very important role in regulating fruit ripening time and delaying fruit softening in small berries such as cherry(Zhai et al., 2022), and kiwifruit (Brian et al., 2021). The fruit of *C. humilis* is also characterized by hard flesh, and therefore is resistant to storage and transportation, which reduces the impact on fruit quality. Fruit ripens and rots quickly at ambient temperature after harvest. Low-temperature storage is a common method of storing and transporting *C. humilis* fruit. Therefore, in this study, we aimed to identify and characterize the *Dof* gene family members of *C. humilis* to determine their on the quality of fruit during post-harvest storage at low temperature. The results of this study will help to improve the quality of *C. humilis* fruit and establish the best method for their storage and transportation.

## 2 Materials and methods

### 2.1 Data acquisition, plant material and treatments

The genome sequence and tissue-specific transcriptome data of *C. humilis* were downloaded from the National Genome Database (https://ngdc.cncb.ac.cn/databases) (Zhao et al., 2022). The amino acid sequences of *Arabidopsis* Dof were downloaded from the *Arabidopsis* Information Resource (TAIR; https://www.arabidopsis.org/). Genome sequences of tomato, *Arabidopsis*, apple, grape, and rice were downloaded from the National Center of Biotechnology Information (https://www.ncbi.nlm.nih.gov). In this study, the fruit of healthy 5-year-old *C. humilis* plants growing in an orchard in Heilongjiang Province, China, was selected as the experimental material. Twenty fruits were washed with distilled water and dried, and randomly divided into four groups (five fruits per group). The collected fruits were subjected to cold storage for 0, 2, 4, 6, and 8 d. The cold-treated samples were divided into two parts, immediately placed in liquid nitrogen, and stored at -80°C until needed for RNA extraction.

### 2.2 Identification and characterization of *ChDof* genes

To identify candidate ChDof family members, a BLAST search of the *C. humilis* genome data was performed with 36 AtDof amino acid sequences (probe) using TBtools (Chen et al., 2020). To identify *ChDof* gene family members using Pfam (http://pfam.xfam.org/search#tabview=tab0) and NCBI – CDD (https://www.ncbi.nlm.nih.gov/Structure/bwrpsb/BWRPSB.Cgi), a hidden Markov model (HMM) profile of the Dof domain (PF02701) was obtained. Duplicate transcripts were removed and the longest transcripts were selected. The physical and chemical properties of ChDof proteins, including isoelectric point, were analyzed using the online analysis software ExPASy ProtParam (http://web.expasy.org/protparam/last accessed on October 15, 2022). To determine their subcellular localization, the amino acid sequences of ChDof proteins were analyzed using the WoLF POSRT online tool (https://wolfpsort.hgc.jp/).

### 2.3 Phylogenetic analysis

Dof protein sequences were used for phylogenetic analysis. Full-length amino acid sequences of 25 ChDofs and 36 AtDofs were aligned using Clustal X. A Neighbor-Joining evolutionary tree was constructed using MEGA7 based on the following parameters: 1000 bootstrap replications, Poisson model, and partwise delection. The phylogenetic tree was further processed with the online software Evolview (http://evolgenius.info/#/)(Subramanian et al., 2019).

### 2.4 Chromosomal location, synteny, and replication analysis

Information on the chromosomal location of *ChDof* genes was obtained from the *C. humilis* genome annotation files, and the distribution of *ChDof* genes on *C. humilis* chromosomes, according to the Poisson model, was determined using TBtools. The Simple Ka/Ks Calculator (NG) tool of TBtools was used to calculate the Ka, Ks, and Ka/Ks values between collinear gene pairs for selection pressure analysis. The MCScanX (Wang et al., 2012) software was used to determine *Dof* gene duplication events both among and within species, including tomato, *Arabidopsis*, apple(*Malus pumila*), grape(*Vitis vinifera*) and rice (*Oryza sativa*), and the results were visualized using TBtools.

### 2.5 Motif sequence and cis-acting element analyses

Motif analysis was performed on the identified *ChDof* gene family members using MEME v5.5.0 (https://meme-suite.org/meme/) (Bailey et al., 2009), based on the following parameters: site distribution, zero or one per sequence; number of motifs:10; and default values for other parameters. Multi-sequence alignment of ChDof was performed using DNAMAN, and the conserved domain of Dof was demonstrated. Using the online Batch CD search (https://www.ncbi.nlm.nih.gov/Structure/bwrpsb/bwrpsb.cgi) to identify ChDof members Domain analysis. The 2000-bp sequence upstream of the ChDof initiation site ATG was extracted from the *C. humilis* genome. Using PlantCARE (http://bioinformatics.psb.ugent.be/webtools/plantcare/html/) (Lescot. et al., 2002) online analytical software conveniently the original sequence of extraction effect analysis, Parameter is the default parameter.

### 2.6 Analysis of *ChDof* gene expression in different tissues

The transcript abundance of *ChDof* genes in roots, stems, leaves, flowers, and fruits was analyzed based on the previously published transcriptome data of *C. humilis*. The tripartite duplicate data of different tissues were averaged and expressed as log2 (FPKM+1) values. A heat map was drawn using TBtools.

### 2.7 RNA extraction and quantitative real-time PCR assays

Total RNA was extracted from the pulp of *C. humilis* fruits using the Plant Total RNA Extraction Kit (Simgen Biotechnology Co., Ltd. Hangzhou, China), according to the manufacturer’s instructions. The quality of total RNA was assessed by electrophoresis on 1% agarose gels and measurement with Nanodrop 1000 spectrophotometer. Then, the total RNA was reverse-transcribed using the SureScriptTM First-Strand cDNA Synthesis Kit (GeneCopoeia, Rockville, MD, USA), and the concentration of the obtained cDNA was determined using a Nanodrop spectrophotometer. Then, to detect ChDof gene expression, qRT-PCR was performed using Q-PCR kit BlazeTaqTM SYBR^®^ Green Qpcr Mix 2.0 and gene-specific primers designed using Primer 3 (**Supplementary Table 2**). The PCR program was as follows: 94 °C for 30 s; 45 cycles of 94 °C for 12 s, 58 °C for 30 s, and 72 °C for 45 s; followed by 79 °C for 1 s for plate reading. After the final PCR cycle, temperature was increased from 55 °C to 99°C at a rate of 0.5 °C per second to generate the melting curve for samples. Actin was used as the internal reference gene. Relative gene expression was calculated using the 2^−ΔΔCt^ method (Livak and Schmittgen, 2001). Each reaction was repeated three times, and the results were expressed as an average of three independent biological replicates.

## 3 Results

### 3.1 Identification of *ChDof* genes

A total of 25 *ChDof* genes were identified based on the whole-genome data of *C. humilis*. Analysis using Pfam and NCBI-CDD databases revealed that all 25 *ChDof* genes contained the Dof domain. *ChDof*s were named as *ChDof1–ChDof25*, according to their chromosomal distribution. The full-length coding sequences (CDSs) of these *ChDof* genes ranged from 489 to 1548 bp, and were predicted to encode 163–516 aa proteins, with a molecular weight ranging from 18.129 to 55.419 kDa and isoelectric point ranging from 4.78 to 9.43. The grand average of hydropathicity varied between -0.482 and -0.937, indicating that the predicted ChDof proteins are hydrophilic. Subcellular localization analysis showed that only ChDof19 localized to the chloroplast, while the other ChDofs localize to the nucleus. Basic details of all members of the ChDof family are summarized in **Supplementary Table 1**.

### 3.2 Phylogenetic analysis and classification of ChDof proteins

**Figure 1:**
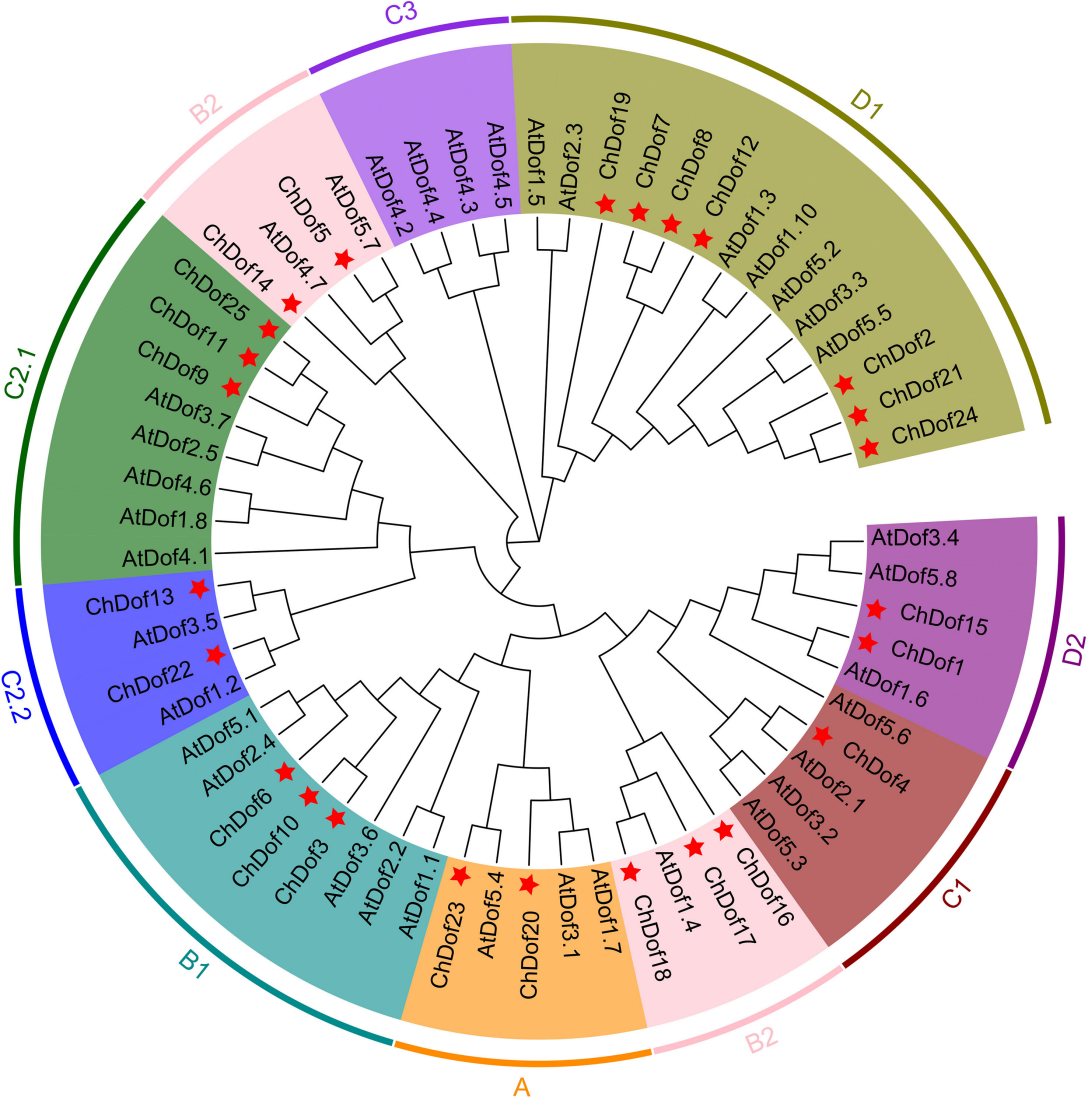
Phylogenetic analysis of Dofs in *Arabidopsis* and *C. humilis* by NJ tree. The tree include 37 Dofs from *Arabidopsis thaliana*, 25 Dofs from *C. humilis*, and construction was based on the full-length protein sequences.

To comprehensively analyze the phylogenetic relationships among ChDof proteins, 36 AtDof proteins and 25 identified ChDof proteins were used to construct a phylogenetic tree (**Figure 1**). According to the previously reported classification of Dof proteins, AtDofs were divided into four evolutionary subgroups (A-D). Among these, subgroup B was further classified into two branches (B1 and B2), subgroup C into three branches (C1, C2.1, and C2.2), and subgroup D subgroup into two branches (D1 and D2). Group A included two ChDof proteins; Group B included eight ChDofs (three in subgroup B1 and five in subgroup B2); Group C contained six members (one in C1, three in C2.1, and two in C2.2); Group D contained nine members (seven in D1 and two in D2). Subfamily C3 contained no ChDof members.

### 3.3 Analysis of conserved motifs and gene structure

**Figure 2:**
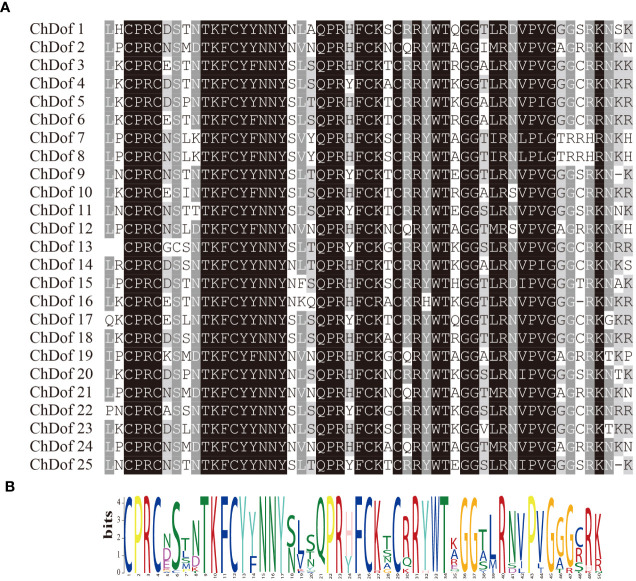
Dof domain sequence alignment of ChDof proteins. **(A)** Extraction of conserved domains of Dof proteins; **(B)** the conserved sequence logo of the Dof domain.

**Figure 3:**
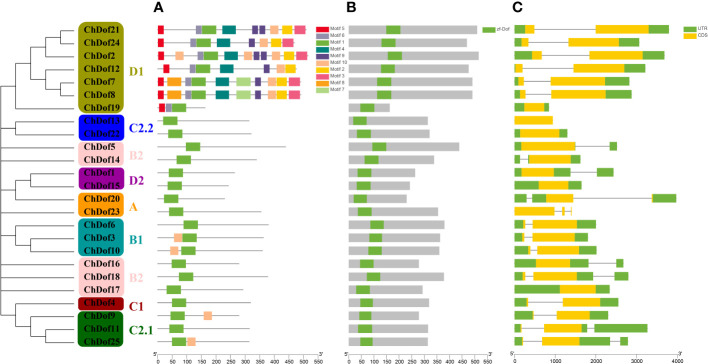
Phylogenetic, gene structure, and conserved motif analysis of *ChDofs*. **(A)** The phylogenetic tree divided *Dofs* into four subgroups. **(B)** Structures of *ChDof* genes. Black rectangles indicate up/down stream sequences; gray rectangles indicate exons; gray lines indicate introns. **(C)** Conserved motif analysis of ChDofs. Different motifs are identified and displayed in different colors.

Ten motifs were predicted in ChDof proteins by MEME. Motif1 was the most important motif, because it was found in all 25 ChDof proteins. Motif1 corresponded to a single zinc finger structure (CX2CX21CX2C) in the Dof domain, and showed a highly homologous core region in this family (**Figures 2A, B**). Only motif1 was found in subfamilies A, B2, C1, C2.2 and D2. Some members of the B1 and C2.1 subfamilies also contained Motif10. In ChDof3 and ChDof10 (B1 subfamily), motif10 occurred before motif1, whereas in ChDof9 and ChDof25 (C2.1 subfamily), motif10 was present after motif1. Interestingly, the D1 subfamily is the most motif-containing subfamily. ChDof7 and ChDof8 contained all 10 motifs, while ChDof19 contained only 3 motifs. Among the 20 motifs, motif1, motif2, motif4, motif5, motif6, motif9, and motif10 were common in the six subfamily members, except ChDof19 (**Figure 3A**). Domain analysis of the 25 ChDof proteins revealed that all domains contained only zf-Dof domains (**Figure 3B**). We analyzed the distribution of introns and exons in *ChDof* genes using TBtools (**Figure 3C**). The results showed that 15 *ChDof* genes contained one intron, 5 ChDofs contained two introns, and 5 *ChDof*s possessed no introns. Most genes in the same group exhibited similar exon-intron structure. For example, most members of Group A and Group C2.1 contained two introns, while most members of subgroup D1 contained only one intron. Moreover, members of subgroup D1 showed a highly similar exon-intron structure and protein localization pattern.

### 3.4 Chromosome location, duplication, and synteny analysis of *ChDof* genes

**Figure 4:**
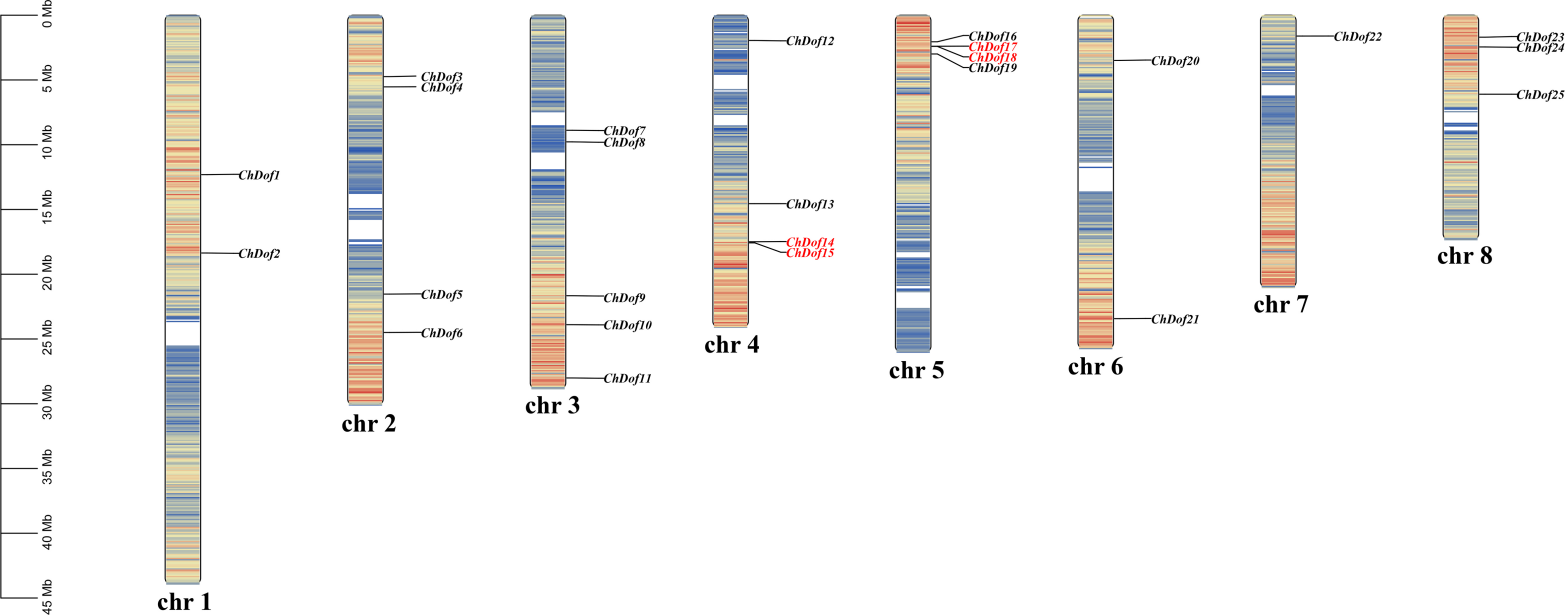
Chromosome location of *ChDof* genes. All 25 *ChDof* genes are shown on the chromosomes and indicated by their names. The left scale represents the length of the *C. humilis* chromosomes. Chromosome numbers are presented at the bottom of each bar. Tandemly duplicated gene pairs are indicated with red.

**Figure 5:**
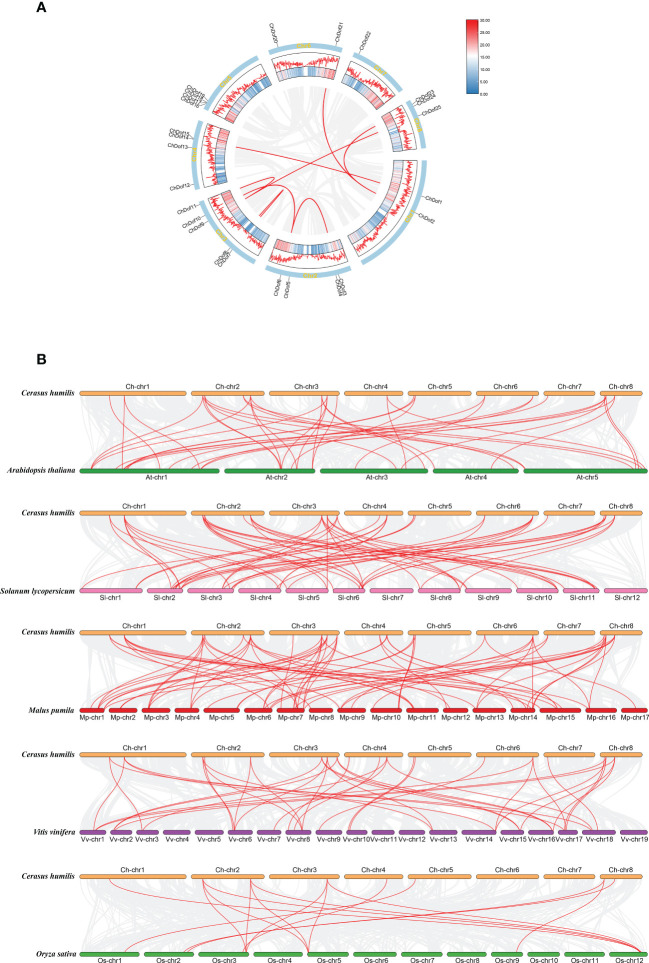
Chromosomal locations and Synthetic analysis. **(A)** Chromosomal locations and segmental duplication events of 25 *ChDof* genes. Red lines represent segmental duplication events. **(B)** Synthetic analysis of *Dof* genes in *C. humilis*, *Arabidopsis thaliana*, *Solanum lycopersium*, *Malus pumila*, *Vitis vinifera* and *Oryza sativa* genomes. The red lines represent homologous gene pairs between two adjacent species.

We determined the distribution of *ChDof* genes in the genome. The 25 *ChDof* genes were unevenly located on eight chromosomes (**Figure 4**). Chromosome 3 (Chr3) harbored the highest number of *ChDof* genes (five), followed by Chr2, Chr4, and Chr5, each of which carried four *ChDof* genes, and *ChDof14* is closely related to *ChDof15*, *ChDof17*, and *ChDof18*. Chr1, Chr6, Chr7, and Chr8 contained 2, 2, 1, and 3 *ChDof* genes, respectively. Duplication analysis revealed two pairs of duplicates: *ChDof4*–*ChDof5* and *ChDof7*–*ChDof8*. Intraspecific collinearity analysis lead to the identification of eight pairs of genes with fragment repetition: *ChDof21*/*ChDof2*, *ChDof1*/*ChDof14*, *ChDof24*/*ChDof2*, *ChDof6*/*ChDof4*, *ChDof6*/*ChDof10*, *ChDof7*/*ChDof8*, *ChDof9*/*ChDof25*, and *ChDof9*/*ChDof11* (**Figure 5A**). These results indicate that fragment replication was the main driving force of *ChDof* gene family evolution. The non-synonymous (Ka) and synonymous (Ks) rates as well as the Ka/Ks ratio are important indicators of species evolution and natural selection. The Ka/Ks values of eight homologous genes were less than 0.5(range: 0.17 -0.45; average: 0.25), indicating that the Dof gene family of *C. humilis* was under strong purification selection and was highly conserved during evolution (**Supplementary Table 3**). To further understand the origin and evolution of *Dof* family genes in different species, a synteny analysis was performed among *C. humilis*, dicots such as *Arabidopsis*, grape, apple, and tomato, and monocots such as rice (**Figure 5B**). A total of 39, 39, 70, 56, and 15 collinear *Dof* gene pairs were identified between *C. humilis* vs. *Arabidopsis*, grape, apple, tomato, and rice, respectively. The number of collinear gene pairs identified between *C. humilis* and apple (Rosaceae) was the highest, and the number of collinear gene pairs between *C. humilis* and rice (monocot) was the lowest. In addition, the number of collinear gene pairs between two dicotyledonous plants was greater than that between two monocotyledonous plants. These results could serve as a valuable gene function reference for Rosaceae cash crops.

### 3.5 Analysis of cis-acting elements in ChDof gene promoters

**Figure 6:**
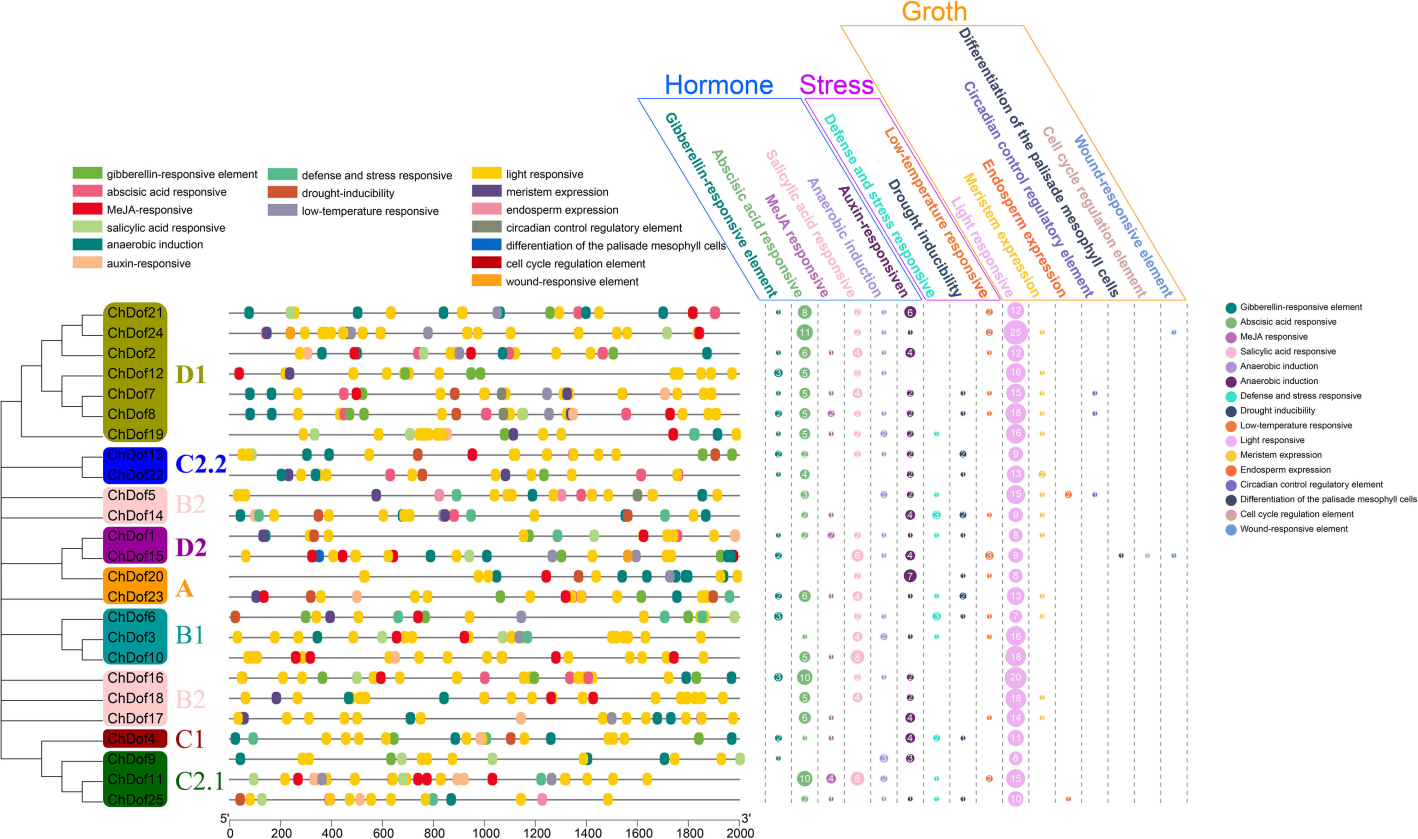
The prediction of a cis-acting element in the 2000 bp promoter upstream of the 25 *ChDof* genes.

To further understand the function of *ChDof* gene family members, cis-acting element analysis was performed on the promoter regions of all 25 genes (**Figure 6**). A total of 680 cis-acting elements were identified, which could be classified into three categories (Hormone, Stress, and Growth). Among the cis-acting elements related to hormone, 16 were involved in gibberellin, ABA, methyl jasmonate (MeJA), salicylic acid (SA), and auxin response, defense and stress response, drought inducibility, low temperature, light, and wound response, anaerobic induction, meristem expression, endosperm expression, circadian control, palisade mesophyll cell differentiation, and cell cycle regulation. Among the cis-acting elements related to growth, 335 (93.05%) were related to photoreaction and were dispersed throughout the promoter regions. Although the total number of cis-acting elements associated with meristem was small, these elements were found in the promoters of 13 *ChDof* genes; the other five growth-related cis-acting elements appeared only in individual *ChDof* gene promoters. Among the cis-acting elements associated with hormones, ABA-responsive elements accounted for the largest number (105, 36.97%). Other hormone-related cis-acting elements were also abundant and were found in the promoters of most members. In abiotic stress, the number of cis-acting elements was close to that of defense stress response, drought and low temperature, and relatively small compared with the total number of cis-acting elements, indicating that the promoters of *ChDof* genes do not play a major role in the abiotic stress response of plants.

### 3.6 Differential analysis of ChDof expression in transcriptome of different tissue

**Figure 7:**
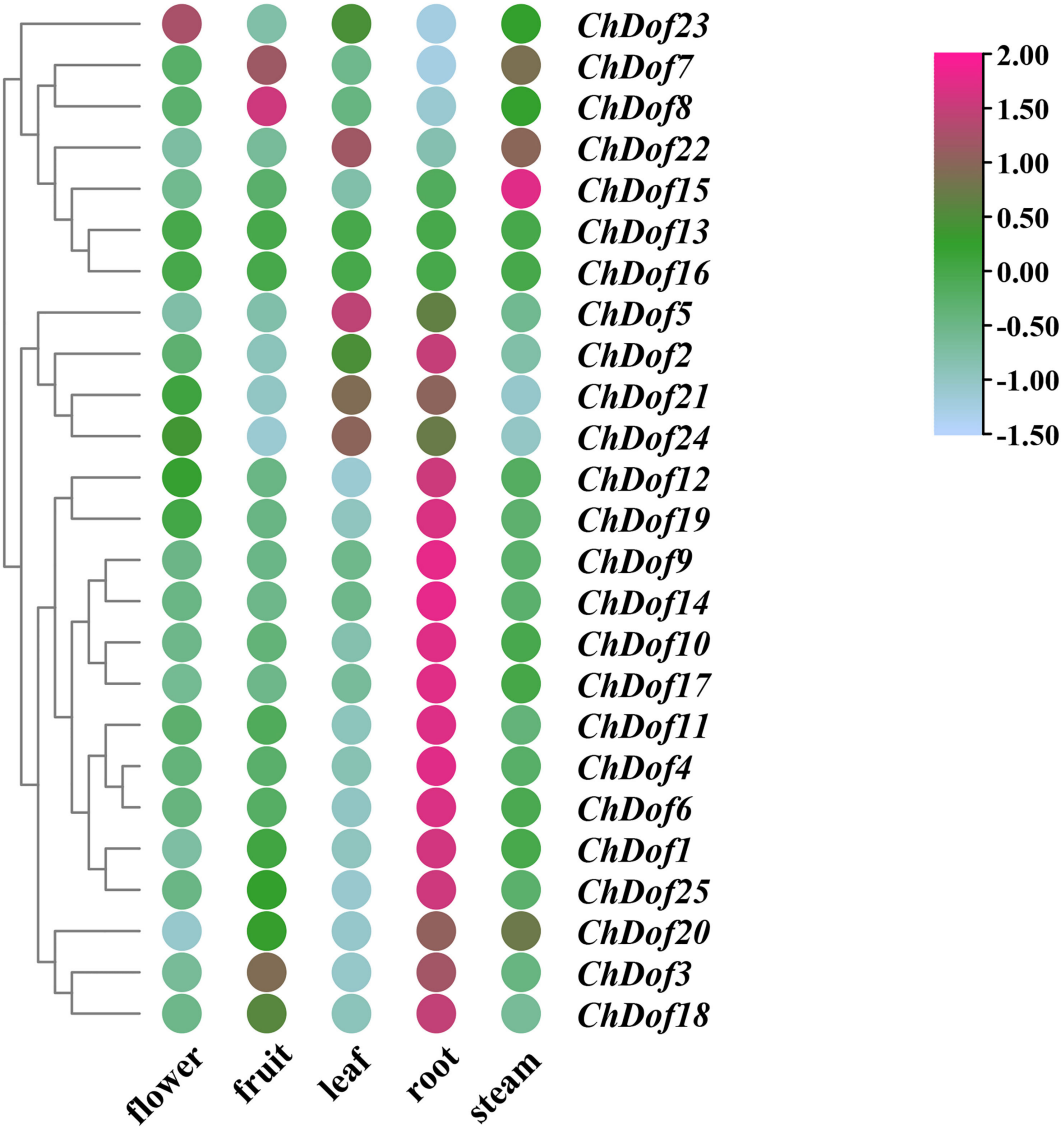
Expression pattern of *ChDofs* in different tissues of *C. humilis*. The expression pattern was generated based on FPKM plus 1 after log2 transformation and analyzed by heatmap hierarchical clustering.

The expression levels of *ChDof* genes in different tissues were analyzed by downloading the transcriptome data of *C. humilis* from National Genome Database. Based on their expression levels, the 25 *ChDof* genes could be classified into three groups (**Figure 7**). Most of the genes in Group1 were highly expressed in the root, while most of the genes in Group2 were highly expressed in the leaf, and their expression levels in other plant parts were low. Genes in Group3 were mainly expressed in flowers, fruits, and stems, and *ChDof13* and *ChDof16* were not expressed in all tissues. The expression of 16 *ChDof* genes was significantly higher in roots than in other tissues. *ChDof23* showed the highest expression level in flowers, *ChDof7* and *ChDof8* in fruits, *ChDof5*, *ChDof22* and *ChDof24* in leaves, and *ChDof15* in stems. This suggests that most members of the *ChDof* gene family act in different tissues, while *ChDof13* and *ChDof16* may be expressed in other tissues or growth and developmental cycles.

### 3.7 *ChDof* gene expression in fruit at different storage times

**Figure 8:**
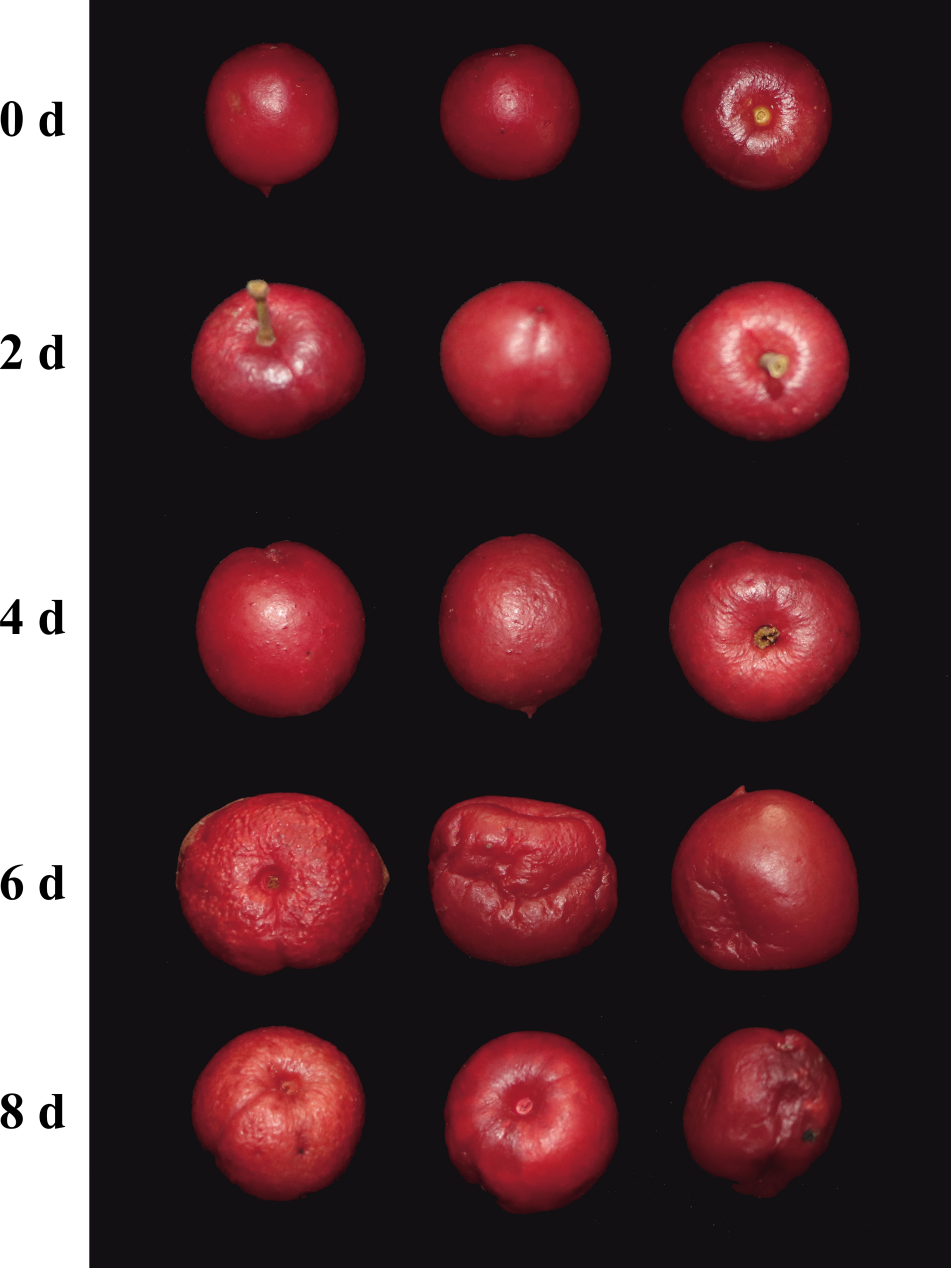
Fruit surface changes with low temperature storage time of *C. humilis*.

**Figure 9:**
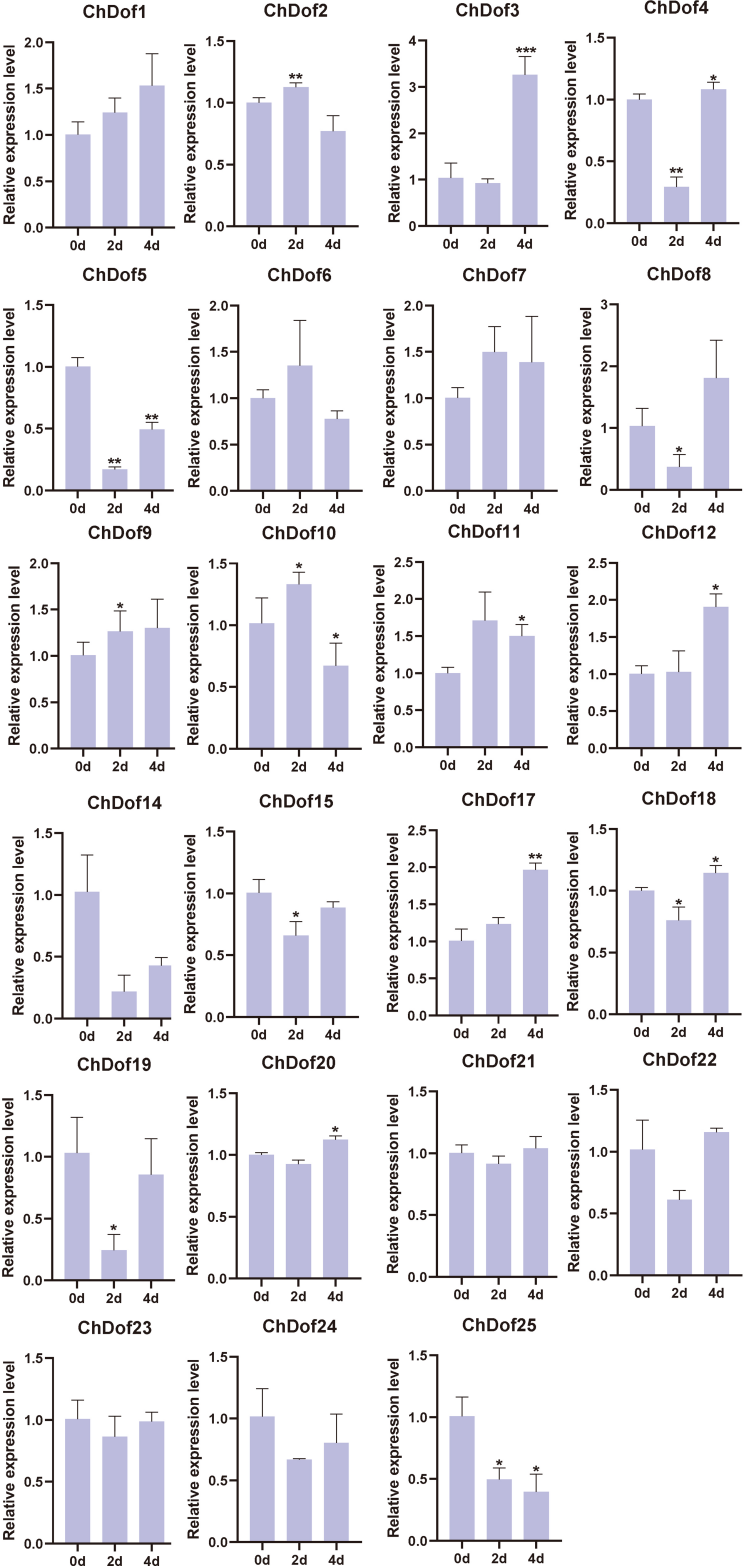
The qRT-PCR analysis of *ChDofs* in response to low-temperature storage. Asterisks indicate statistically significant differences compared with the 0d (Student’s t-test: *p < 0.05; **p < 0.01; ***p < 0.001).

The comparison of *C. humilis* fruit stored at 4°C for 0, 2, 4, 6, and 8 d showed that the surface of *C. humilis* fruits was wrinkled, and the severity of these wrinkles increased with storage time (**Figure 8**). RNA was extracted from *C. humilis* at five time points. Gel electrophoresis showed that RNA degraded with the extension of storage time, and no RNA could be extracted from *C. humilis* fruits sampled at 6 and 8 d (**Figure S2**). Therefore, gene expression could be analyzed only in *C. humili*s fruit sampled at 0, 2, and 4 d (**Figure 9**). The results of qRT-PCR showed that the expression profiles of *ChDof1*, *ChDof3*, *ChDof7*, *ChDof9*, *ChDof11*, *ChDof12*, and *ChDof17* were similar and tended to increase with storage time; the expression of *ChDof2*, *ChDof6*, *ChDof10*, and *ChDof25* first increased and then decreased; the expression of *ChDof4*, *ChDof8*, *ChDof19*, *ChDof22*, and *ChDof24* first decreased and then recovered; the expression profiles of *ChDof5* and *ChDof25* showed a decreasing trend; and the expression of *ChDof15*, *ChDof18*, *ChDof20*, *ChDof21*, and *ChDof23* showed little change. Additionally, the expression levels of *ChDof13* and *ChDof16* were equal to 0 in different storage time nodes. These results indicate that the expression levels of *ChDof* genes are almost independent of fruit development, consistent with the results of transcriptome analysis.

## 4 Discussion

The fruit of *C. humilis* can not only be consumed fresh but can also be made into wine, juice, jam, candy, and other products. *C. humilis* fruits are popular for their high calcium content compared with other fruits of the same type. Moreover, because of their high flavonoid content, *C. humilis* fruits can be used as an effective natural antioxidant for preventing free radical-induced damage to the human body to varying degrees, and thus have high application potential in the healthcare industry. Post-harvest storage has a considerable impact on fruit quality. Therefore, it is important to mine and analyze the transcription factors related to fruit quality during storage. *Dof* genes are prevalent in all plant species and play a vital role in various physiological processes. No studies have been conducted on *Dof* genes in C. humilis to date. In this study, we conducted a comprehensive analysis of *ChDof*s, focusing on changes in their expression in fruit during storage. The results of this study help us further understand how *ChDof* genes work and how to improve the storage environment of *C. humilis* fruit.

In this study, whole-genome analysis revealed 25 *ChDof* genes in *C. humilis*, which were divided into eight subfamilies (A, B1, B2, C1, C2.1, C2.2, D1, and D2) based on their phylogenetic analysis with AtDofs (**Figure 1**). These genes were named as *ChDof1–ChDof25*, according to their chromosomal location. Different number of *Dof* genes have been reported in plant species; for example, 28 in *Eugenia uniflora* (Waschburger et al., 2022), 22 in spinach (*Spinacia oleracea*) (Yu et al., 2021), 16 in tea (*Camellia sinensis*) (Yu et al., 2020), 45 in pear (*Pyrus bretschneideri*) (Liu et al., 2020), 24 in rose (*Rosa chinensis*), 40 in *Medicago sativa*(Cao et al., 2020), 25 in grape, 36 in watermelon (*Citrullus lanatus*) (Zhou et al., 2020), 36 in *Areca catechu* (Li et al., 2022a), and 20 in *Chrysanthemum morifolium* (Song et al., 2016). However, some plant species possess an exceptionally high number of *Dof* genes (e.g., 103 in *Camelina sativa*) (Luo et al., 2022), which may be related to the large genome size or high sequencing depth of the species. *Dof* genes were divided into nine subfamilies in Tartary buckwheat and watermelon(Zhou et al., 2020; Li et al., 2022b), but only into eight subfamilies (no C3 subfamily) in *C. humilis*, which may be because of gene loss in the evolutionary process. Only seven subfamilies of *Dof* genes have been identified in lotus. In rice and sorghum, at least 11% of *Dof* genes disappeared between 70 and 50 million years ago (MYA), because of changes in amino acid substitution rates in the critical Dof domain (Yu et al., 2021). This confirms that the deletion of *Dof* gene family members is common among plant species.

Gene structure and motif distribution provide supporting evidence for the evolutionary relationships among species or genes. Analysis of amino acid sequences revealed that ChDof proteins contained highly conserved Dof domains (**Figure 2**), indicating that the evolution of Dof transcription factors in plants is conserved. In addition to the Dof domain, nine different motifs, with differential distribution among ChDofs, were found. Intron-exon differentiation is closely related to plant evolution, and often members of the same subfamily have similar exon-intron structure and number distribution. Therefore, the genetic diversity of *ChDof* introns and exons is the basis of the evolution of polygenic families. The number of introns in *ChDof* genes varied from 0-2, which is similar to the number of introns in the *Dof* genes of pepper (0–2) (Kang et al., 2016; Wu et al., 2016), sorghum (0–2) (Kushwaha et al., 2011), and banana (0–4) (Dong et al., 2016). This indicates that the *ChDof* gene structure is relatively stable.

Cis-acting elements play a crucial role in gene expression, and the study of gene promoters is essential for understanding the general control of plant gene expression. In this study, many elements were found in the *ChDof* gene promoters. Among these elements, those involved in the response to plant hormones including ABA, biotic stress, abiotic stresses including low temperature and light, and plant growth and development response were the most abundant (**Figure 6**). These results are very similar to the results of cis-acting element analysis in lotus, indicating that the composition of Dof gene promoters is also relatively conserved (Cao et al., 2022). Storage or transport at low temperature is commonly used to extend the shelf-life of fruits and vegetables after harvest. However, low temperature can cause chilling injury, resulting in the deterioration of fruit quality and a severe decline in commodity value. A previous study showed that ABA could improve the chilling tolerance of peach fruit by inhibiting the production of ethylene and promoting the scavenging ability of hydrogen peroxide (H_2_O_2_) (Tang et al., 2022). In our study, we discovered that *ChDof* gene promoters harbored several ABA-responsive cis-acting elements. Therefore, application of ABA in low temperature environment regulated the expression of *ChDof* genes to reduce the damage to fruit caused by cold injury.

The analysis of differentially expressed genes indicated that these genes may be involved in plant growth and development (**Figure 7**). *ChDof23*, *ChDof8*, *ChDof5*, and *ChDof15* were highly expressed in flowers, fruits, leaves, and stems, respectively. Most Dof genes are specifically overexpressed in the root. *ChDof13* and *ChDof16* were not expressed in any of the five tissues, suggesting that these two genes may be pseudogenes or expressed only under special conditions(Dong et al., 2016). Most Dof genes play a critical role in plant signaling and regulatory networks associated with abiotic/biotic stress responses and many developmental/physiological processes. A previous study in grape showed that *Dof* genes are involved in different stages of the fruit growth and ripening process (da Silva et al., 2016). In addition, fruit quality is closely related to seed size, and seed, as the main source of tannins produced by fruit, directly affects the production and quality of fruit wine. Some *Dof* genes showed significant transcript accumulation at all stages of seed growth and development (da Silva et al., 2016). Dof transcription factors also regulate flowering time. Overexpression of the *PbDof 9.2* gene in *Arabidopsis thaliana* inhibited the expression of *FLOWERING LOCUS T* (*FT*), which regulates flowering time, by promoting the activity of *PbTFL1a* and *PbTFL1b* promoters, leading to delayed flowering (Liu et al., 2020). *Dof* genes are also involved in the regulation of plant secondary metabolites. Transient overexpression of *FcDof4* and *FcDof16* enhanced the transcription of structural genes in the flavonoid biosynthesis pathway and increased the content of C-glycosyl flavonoids. Additionally, it was demonstrated that FcDof4 and FcDof16 transcription factors promote the synthesis of flavonoids in kumquat fruits by activating the expression of FcCGT (Yang et al., 2022). It has important guiding significance for further study on the regulation of *ChDof* genes on the biosynthesis of flavones in *C. humilis*. To explore the potential functions of *ChDof* genes in response to cold storge, we analyzed the expression of 25 *ChDof* genes by RT-qPCR (**Figure 9**). The expression profiles of most *ChDof* genes changed over time, suggesting that these genes affect the quality of *C. humilis* fruit during storage. Some *ChDof* genes, which were not expressed, might exhibit other functions or may be expressed only under special conditions.

## 5 Conclusion

This study is the first to comprehensively analyze the *Dof* gene family members in *C. humilis*. A total of 25 *ChDof* genes were identified in the *C. humilis* genome, which were distributed on eight chromosomes and were divided into eight subgroups. Conserved zinc finger domains (C2-C2) were found in all ChDof proteins, and fragment replication was found to be the main driving force of ChDof evolution. In addition, *ChDof* genes showed tissue-specific expression patterns. Additionally, the expression of most *ChDof* genes in fruit changed during storage at 4 °C, which suggests that these genes are involved in the regulation of fruit quality after picking. Overall, this study provides a strong foundation for further research on the functional characteristics of *ChDof* genes, especially their role in fruit quality regulation during postharvest storage.

## Data availability statement

The datasets presented in this study can be found in online repositories. The names of the repository/repositories and accession number(s) can be found here:

tomato GCF_000188115.4 https://www.ncbi.nlm.nih.gov/assembly/GCF_000188115.4/
Arabidopsis GCA_000001735.4 https://www.ncbi.nlm.nih.gov/assembly/GCF_000001735.4/
apple GCF_002114115.1 https://www.ncbi.nlm.nih.gov/assembly/GCF_002114115.1/
grape GCF_000003745.3 https://www.ncbi.nlm.nih.gov/assembly/GCF_000003745.3/
rice GCA_000005425.2 https://www.ncbi.nlm.nih.gov/assembly/GCF_000005425.2/.

## Author contributions

YWL, and WM designed the experiments. WL, WR, and XL carried out the experiments. WL, CQ, PW, and LH analyzed the experimental results. WR, LK, and YL wrote the manuscript. All of the authors have approved the final manuscript.

## Supplementary material

Supplementary Figure 1 The legend depicting the protein sequence of the corresponding motif by MEME analysis.

Supplementary Figure 2 Agarose gel electrophoresis of RNA extraction from mature calcium fruit stored at 4°C for different days.

Supplementary Table 1 Characterization of 25 *ChDof* transcription factors, and Summary of ChDof sequences.

Supplementary Table 2 The primers sequences used to determine the genes expression levels of *ChDof* genes by qRT-PCR.

Supplementary Table 3 Ka/Ks data analysis.
