# Supplementary figures and images for: Identification and characterization of Dof genes in Cerasus humilis
Source: Front Plant Sci. 2023 Apr 3;14:1152685. doi: 10.3389/fpls.2023.1152685 (PMC10106723; doi:10.3389/fpls.2023.1152685)

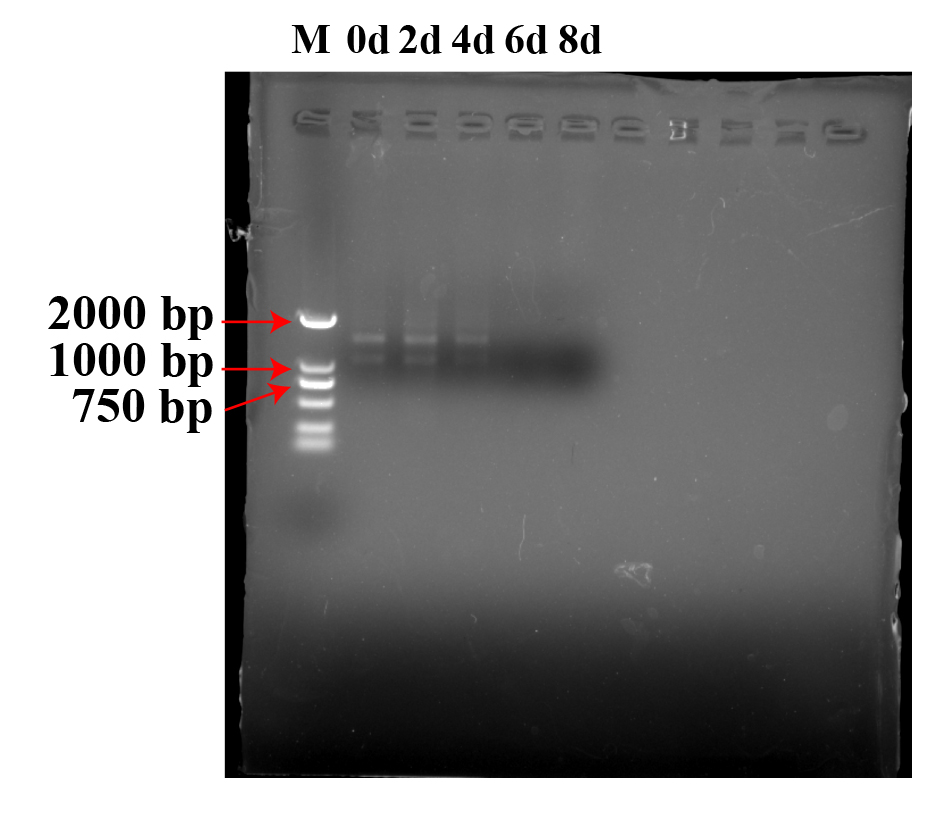

Supplement: Supplementary Figure 2 — Agarose gel electrophoresis of RNA extraction from mature calcium fruit stored at 4°C for different days. [file Image_2.jpg]
